# Person Re-Identification with Attribute-Guided, Robust-to-Low-Resolution Drone Footage Considering Fog/Edge Computing

**DOI:** 10.3390/s25061819

**Published:** 2025-03-14

**Authors:** Bongjun Kim, Sunkyu Kim, Seokwon Park, Junho Jeong

**Affiliations:** Department of Computer Science and Engineering, Dongguk University, Seoul 04620, Republic of Korea; rprprp01@gmail.com (B.K.); code@dgu.ac.kr (S.K.); rty3394@dongguk.edu (S.P.)

**Keywords:** re-identification, vision, fog/edge computing, drone footage, aerial surveillance, eXplainable Artificial Intelligence (XAI)

## Abstract

In aerial surveillance using drones, person re-identification (ReID) is crucial for public safety. However, low resolutions in drone footage often leads to a significant drop in ReID performance of subjects. To investigate this issue, rather than relying solely on real-world datasets, we employed a synthetic dataset that systematically captures variations in drone altitude and distance. We also utilized an eXplainable Artificial Intelligence (XAI) framework to analyze how low resolutions affect ReID. Based on our findings, we propose a method that improves ReID accuracy by filtering out attributes that are not robust in low-resolution environments and retaining only those features that remain reliable. Experiments on the Market1501 dataset show a 6.59% percentage point improvement in accuracy at a 16% resolution scale. We further discuss the effectiveness of our approach in drone-based aerial surveillance systems under Fog/Edge Computing paradigms.

## 1. Introduction

**Figure 1:**
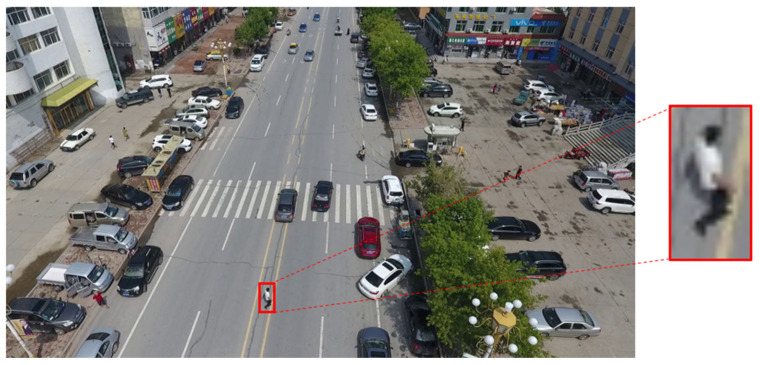
Example of human imagery in drone footage.

In order to maintain effective urban security, fixed CCTV systems have been widely adopted in the field of video surveillance, and the number of such installations continues to grow [1]. However, a major limitation of conventional fixed CCTV systems is their inability to track and identify a specific target, coupled with blind spots arising from their restricted fields of view. To address these shortcomings, the use of drones in tandem with advanced person re-identification (ReID) technologies has recently emerged as a promising alternative for aerial surveillance, and numerous practical applications have been reported. However, current deep-learning-based ReID frameworks are predominantly designed for footage captured by low-angle, fixed-position cameras (CCTVs). As a result, when applied to drone-captured images, they suffer from substantially reduced accuracy, largely due to low capture resolutions and distortions exacerbated by altitude and distance between the drone and the subject (see Figure 1).

Recently, ReID research has progressed rapidly, driven by the appearance of large-scale public datasets such as Market1501 [2], MSMT17 [3], and VisDrone [4]. Nonetheless, most ReID models are trained using images collected from fixed camera viewpoints. Consequently, they tend to perform poorly under high-altitude, wide-field-of-view scenarios, like those often encountered in drone footage, where resolution is greatly reduced [5,6]. Although various approaches, including Super-Resolution (SR) techniques [7,8], have been proposed to enhance the resolution of low-resolution images and thus improve ReID accuracy, such methods often impose substantial computational overheads, potentially rendering them impractical for real-time scenarios.

Meanwhile, to make drone-based aerial surveillance more viable, it has become crucial to determine how and where to process the large volumes of low-resolution video data collected by drone sensors. Fog/Edge Computing has attracted considerable attention as a promising paradigm, because it allows for filtering, preprocessing, and simple inference at the Edge or Fog Nodes rather than transmitting all data to a central cloud server, thereby reducing network load and enabling real-time processing [9,10]. Recently, a study combining wireless power transfer (WPT) with Multi-Access Edge Computing (MAEC), leveraging deep reinforcement learning (DRL) to minimize offloading latency, has been reported [5], further broadening the scope of Edge/Fog Computing.

Accordingly, this study aims to analyze the problem of ReID performance degradation caused by low resolution more effectively by generating synthetic human-image data that quantitatively accounts for changes in resolution according to drone altitude and distance. We use Epic Games’ Unreal Engine 5 [11], known for its high-quality graphics, along with Microsoft’s open-source simulator AirSim [12], to produce the synthetic data.

To improve ReID performance in drone-based aerial surveillance systems operating in tandem with Fog/Edge Computing, we propose a filtering method that leverages attributes robust to low resolutions. Specifically, to collect data covering a range of drone-target distances and angles, we generate synthetic datasets using Unreal Engine 5 [11] and AirSim [12]. We then employ eXplainable Artificial Intelligence (XAI) [13,14,15] to identify which human attributes, such as upper- and lower-body colors, remain robust in low-resolution images, and we develop a filtering strategy that exploits these robust attributes to enhance ReID performance in low-resolution conditions. By applying the same attribute-based filtering approach to a real-world dataset (Market1501 [2]), we confirm that the proposed method can yield a 6.59 percentage point improvement in Rank-1 accuracy at 16% resolution. Our key contributions are as follows:

Use of synthetic data: We construct a quantitative scenario that systematically controls resolution degradation according to drone altitude and distance, going beyond the limitations of existing low-resolution datasets.

Proposal of an attribute-based filtering technique: We focus on human attributes (such as upper-body and lower-body color) that remain robust in low-resolution environments to reduce the number of comparison candidates, thus improving real-time performance.

Strategies for integration of Fog/Edge Computing: To mitigate latency arising from the physical distance between the drone and the control server, we perform attribute-based filtering and partial ReID inference on Edge Nodes, thereby reducing both network traffic and computational load.

Quantitative validation: We verify that the ReID performance improvements demonstrated with our synthetic data also lead to practical gains in the Market1501 [2] dataset.

The remainder of this paper is organized as follows. In Section 2, we review the latest person ReID technologies, prior studies aimed at improving ReID in drone imagery, and both real and synthetic datasets used for training and testing ReID models. Section 3 describes our proposed methodology for enhancing ReID performance in drone imagery. Section 4 discusses the composition of our experimental data, presents the results of our experiments, and analyzes the effectiveness of the proposed approach. Section 5 outlines our strategies for integrating Fog/Edge Computing into the proposed method. Finally, Section 6 suggests avenues for future research, and Section 7 concludes the paper.

## 2. Related Work

In this section, we first explore research trends in low-resolution ReID, and then discuss the challenges of applying major ReID datasets to low-resolution settings and explain the rationale and limitations of employing synthetic data. We also examine approaches that combine Fog/Edge Computing for real-time ReID in drone-based surveillance.

### 2.1. Low-Resolution Re-Identification Research

Traditionally, person ReID has been developed with a focus on ground-level, fixed-camera perspectives [6,16,17]. In environments like drone-based surveillance, however, where camera altitude is high, and the viewing angle frequently changes, the captured individual often appears at a considerably lower resolution. Consequently, existing models suffer a sharp performance drop. To overcome this issue, recent research has begun to focus on cross-resolution ReID and specialized methods for low-resolution images.

Although Super-Resolution (SR) approaches [8,18] aim to restore low-resolution images to boost ReID performance, their heavy computational requirements make them challenging to apply in real-time scenarios. Munir et al. [19] separated images into different scales (downsampled by varying factors) and employed a feature-distillation technique to narrow the gap between low- and high-resolution images, thereby improving cross-resolution ReID. Nonetheless, additional research was needed to fully address dynamic resolution changes and real-time constraints in drone surveillance. CRRCD [20] used relationship-centric contrastive learning to refine feature mapping for low-resolution object recognition, yet it also did not fully account for the extreme resolution degradation found in drone scenarios.

Meanwhile, some studies [14,15] have sought to explain ReID results based on human attributes: rather than simply relying on a scalar distance between person images, they decompose the distance according to detailed human attributes via an interpreter module, thus generating explainable ReID outcomes. By adjusting attribute-based weighting and applying distillation, they improved ReID performance. However, similar to methods that address viewpoint changes by knowledge distillation from multiple views [21], these approaches still focus predominantly on ground-level perspectives and therefore need additional refinements for degraded performance in aerial (drone) images.

### 2.2. Limitations of Existing Re-Identification Datasets

Deep-learning-based person ReID has advanced rapidly, thanks in large part to large-scale datasets such as Market1501 [2] and MSMT17 [3,6]. However, because these datasets were primarily collected from fixed ground-camera perspectives, they do not directly incorporate the low resolutions and extreme viewing angles present in drone imagery. Below are some of the widely used ReID datasets, along with the challenges they pose for drone-based person ReID research:

Market1501 [2]: One of the most commonly used large-scale benchmarks, containing 32,668 images of 1501 pedestrians captured by six stationary cameras. Despite its popularity, it lacks data representing high-altitude or wide viewing angles, thus failing to capture resolution or perspective variations typically seen in drone-based footage.
MSMT17 [3]: Collected from 15 cameras (both indoor and outdoor), comprising 4101 identities and 126,441 bounding-box images. Although it spans various lighting conditions, weather, and times of day, it still mainly relies on stationary or ground-level cameras and thus does not reflect the substantially reduced scale of people photographed from high-altitude drone viewpoints.
VisDrone [4]: A large-scale dataset intended primarily for object detection and tracking in drone videos. It contains labels for pedestrians, vehicles, motorcycles, and so on, but the identity labels required for ReID are limited, and person-attribute annotations are scarce.
TinyPersons [22]: Focuses mainly on “tiny object detection” by providing drone-captured images in which people appear extremely small (approximately 20 × 32 pixels or less). Although it offers 72 K object annotations, it does not specifically include the identity labels necessary for ReID.

While TinyPersons [22] does address extremely small person sizes, it is designed more for object detection than for person ReID, and VisDrone [4] similarly centers on detection/tracking rather than identity-level labels. Hence, these datasets are difficult to use directly for ReID tasks.

### 2.3. Synthetic Data: Rationale and Limitations

Collecting large-scale data under various drone–subject distances and altitudes can be expensive in terms of equipment, manpower, and time, and it can also be challenging to systematically control experimental conditions. To circumvent these issues, synthetic data have gained traction in ReID research. For instance, WePerson [23] and UnrealPerson [24] leverage game engines (e.g., GTA5, Unreal Engine) to automatically generate human characters, reducing labeling costs and improving ReID performance via domain adaptation [25]. Nonetheless, due to differences in texture and optical characteristics collectively known as the domain gap [26] post-processing or additional training may be required.

(1) Real-World Collected Datasets
  Advantages: Minimal domain gap, accurately reflecting real-world conditions and thus minimizing discrepancy between training and testing.
  Disadvantages: Difficult to obtain fine-grained labels (e.g., camera parameters related to altitude and distance). Many real datasets are not designed for ReID, making it challenging to analyze low-resolution performance quantitatively. Annotation costs can also be high.
(2) Synthetic Datasets
  Advantages: Camera parameters such as distance, altitude, and viewing angle can be quantitatively controlled, making it easier to analyze performance under varying resolutions. Privacy concerns are eliminated, and images can be generated in large quantities at will.
  Disadvantages: Domain gap may arise due to differences in textures, lighting, noise, and lens effects compared to real-world images. Additional domain adaptation or mixed training with real data may be necessary for better generalization to real-world scenarios.

In this study, we employ synthetic data primarily to simulate resolution degradation step by step as drone–subject distance and altitude increase, allowing us to experimentally determine which human attributes remain reliably identifiable in low-resolution conditions. Generating such controlled data with real drones would require extensive equipment, manpower, and time, with limited control over labeling quality, lighting, and weather. By contrast, synthetic environments can be freely adjusted.

Using Unreal Engine 5 and AirSim, we replicate a drone’s viewpoint and automatically capture images at different angles and distances, systematically creating low-resolution scenarios. We then analyze attribute recognition accuracy and ReID performance on these synthetic images. Finally, we validate our proposed method on a real-world dataset (Market1501 [2]) to assess its practical applicability, confirming that similar performance improvements can be achieved in a real-world context.

### 2.4. Fog/Edge Computing for Aerial Re-Identification

Fog computing extends cloud capabilities to the network edge, provisioning computing and storage resources closer to end devices [9]. This paradigm is particularly valuable for aerial surveillance using drones, as real-time analytics on high-bandwidth video can be offloaded to Edge Nodes [27]. However, there remains a fundamental limitation to improving ReID accuracy if the extreme resolution degradation inherent in drone footage is not directly addressed [28]. In this paper, we discuss an approach for reducing network and computational burdens while improving ReID accuracy by filtering out irrelevant candidates at Edge/Fog Nodes, using attributes robust to low-resolution (e.g., shirt color and pants color).

When drones capture wide-area footage from high altitudes, the resulting person images are extremely small, making traditional ReID approaches significantly less accurate [28]. By performing initial attribute extraction and candidate filtering at the edge, only relevant bounding boxes need to be transmitted to a remote server, thereby reducing both bandwidth usage and central computational load [27]. This aligns with the original tenet of fog computing—processing data locally to minimize backhaul overhead [9].

Furthermore, distributed learning at Edge Nodes can enhance this system. Federated Learning allows multiple edge devices to train ReID models collaboratively while keeping raw data local, addressing data privacy and transmission concerns [10]. Additionally, within the realm of Wireless Powered Multi-access Edge Computing (WP-MEC), recent work has introduced methods to minimize total energy provision. For instance, Liu et al. [29] propose a multi-HAP (Hybrid Access Point) WP-MEC framework using a two-tier deep reinforcement learning approach, reducing long-term energy consumption while maintaining performance. In single-HAP systems, Li et al. [30] use an ADMM-based distributed algorithm to achieve near-optimal power minimization. Both illustrate that distributing computation intelligently at the network edge can cut down energy usage, which is critical for battery-limited drones.

Meanwhile, Zheng et al. [5] demonstrate that a DRL-based offloading algorithm can drastically lower computation delays in WP-MEC systems, highlighting the power of AI-driven policies for dynamic network environments. Similarly, He et al. [31] propose a cooperative approach to energy-efficient task offloading in wireless-powered MEC, ensuring stable performance under varying loads. These approaches confirm that by assigning tasks between the drone, Edge/Fog Nodes, and possibly a central server, one can overcome the inherent resolution challenge in aerial ReID while also optimizing energy and latency. In this paper, we discuss an approach for reducing network and computational burdens while improving ReID accuracy by filtering out irrelevant candidates at Edge/Fog Nodes, using attributes robust to low-resolution (e.g., shirt color and pants color).

## 3. Proposed Method for Improving Low-Resolution Performance Through Hypotheses and Attribute Filtering

In this section, we hypothesize that certain attributes recognizable even at low resolutions can be effectively identified, and we validate this hypothesis through experiments. We then propose a method to enhance ReID performance by filtering based on these attributes. To test our hypothesis, we describe the process of creating and verifying synthetic data in a manner similar to previous studies [14,15] that annotate fine-grained attributes in the Market1501 [2] dataset.

### 3.1. Generation of Synthetic Data

**Figure 2:**
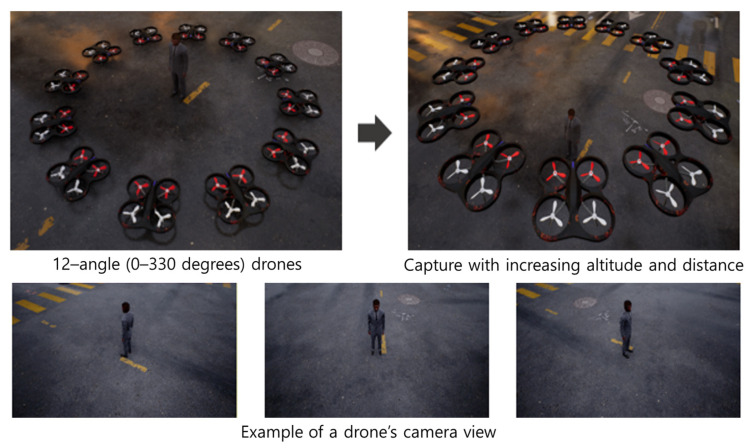
Example of synthetic data generation in the proposed framework.

Existing low-resolution ReID datasets often fail to adequately reflect the varying resolutions caused by changes in distance between a drone and its subject (the person). To address this limitation, we employ the AirSim simulator within the Unreal Engine 5 environment to generate synthetic drone footage at various resolutions. The data-generation process consists of the following three steps:

Virtual Environment Setup and Video Capture: As shown in Figure 2, we first use the MetaHuman creation feature provided by Unreal Engine 5 to generate human subjects. Then, using the AirSim simulator, we position the drone at 12 different horizontal angles (from 0° to 330° at 30° increments) around the individual, so that the drone’s camera faces the person’s body. We systematically adjust the distance between the person and the drone and repeatedly capture images at each configuration, thus creating aerial images under different resolution conditions.

**Figure 3:**
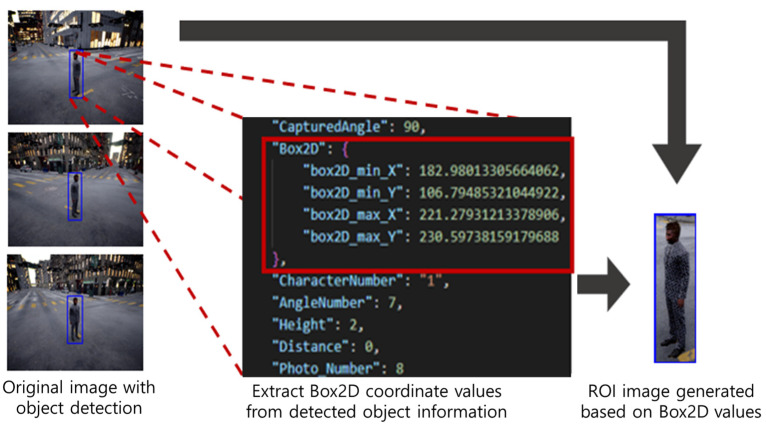
Human region extraction process.

**Figure 4:**
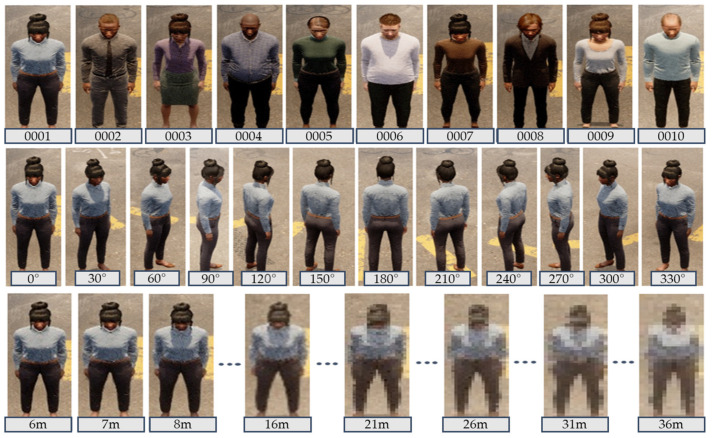
Capturing people areas with object detection.

Human Region Extraction: As depicted in Figure 3, object detection or 2D coordinate projection is used to obtain the coordinates of the person’s region of interest (ROI). Based on these coordinates, we crop each person’s region from the drone images. We then label the resulting cropped image filenames to record parameters such as camera angle, distance, and altitude. As a result, for each individual, we can generate images with various resolutions, captured at 12 angles, with multiple altitudes and distances (Figure 4).

Personal Attribute Labeling: Following earlier studies on annotating human attributes in Market1501 and the Attribute-Guided Metric Distillation (AMD) method [15] for explainable ReID, we manually labeled attributes for each virtual person ID, excluding the binary-labeled “age” attribute from the 27 used in prior work. (See Table 1). Table 2 summarizes the synthetic dataset generated by this approach.

### 3.2. Experiments on Attribute Recognition at Low Resolution Using Synthetic Data

To implement the proposed attribute-based filtering for ReID, we analyze how person ReID accuracy and corresponding attribute recognition results vary with resolution (i.e., changing distances in drone imagery). Our expectation is that while ReID model performance will degrade as distance (and thus resolution) worsens, certain attributes that are robust to low resolution will maintain a relatively stable recognition rate.

For attribute recognition, we use Attribute-Guided Metric Distillation (AMD) [15], an explainable ReID method that extracts attribute information from person images. Our experimental dataset comprises the attribute-labeled Market1501 [2,14] combined with our synthetic dataset. As gallery data, we use images from the synthetic dataset captured at the closest distance (6 m), yielding the highest resolution. For query data, we select images of a particular individual from the synthetic dataset at three levels of resolution: original (high resolution), intermediate resolution, and low resolution. For evaluation metrics, we use the commonly adopted Rank-k and mAP (mean Average Precision) measures in ReID. The ReID and attribute recognition metrics are calculated as follows:

Re-identification (mAP with Rank-50);

1. For each query, rank all gallery images in ascending order of feature distance (ranking from 1 to N).
2. Identify the positions in the ranking where the same ID images appear, and compute Average Precision (AP) by accumulating precision at each of these ranks.
3. The mean of the AP values across all queries is mAP.
4. If needed, calculate Rank-k accuracy (e.g., Rank-1, Rank-5, Rank-10).

Attribute recognition (mAP with Rank-num of same attributes);

1. Based on the attribute information for each query-gallery pair, compute an attribute-wise distance.
2. For each pair, determine how many attributes are actually identical (num_same) and then, in the ranked list of attributes, treat the top num_same as ’same’.
3. In this attribute-based ranking, each time an attribute that truly matches (“same”) appears, accumulate the precision to calculate the Average Precision (AP). Finally, the mean AP across all pairs is taken as the mAP.

**Figure 5:**
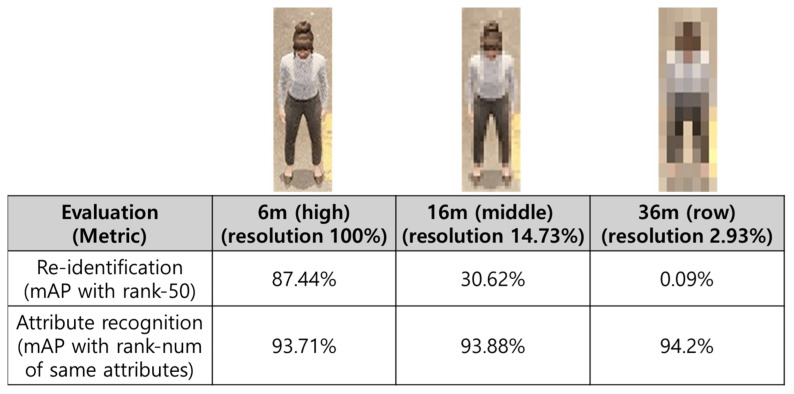
Re-identification accuracy and attribute recognition results by resolution.

As summarized in Figure 5, the experimental results illustrate how ReID metrics change under increasing drone–subject distance (hence lower resolution). We observe a substantial drop in ReID accuracy as resolution decreases. At a 10% resolution level, the mAP is a mere 0.09%. By contrast, the human attributes measured indirectly from feature values between queries and galleries remain relatively stable, suggesting that certain attributes are indeed robust against resolution degradation.

### 3.3. Experiments on Low-Resolution Personal Attribute Recognition Using Real Data

**Figure 6:**
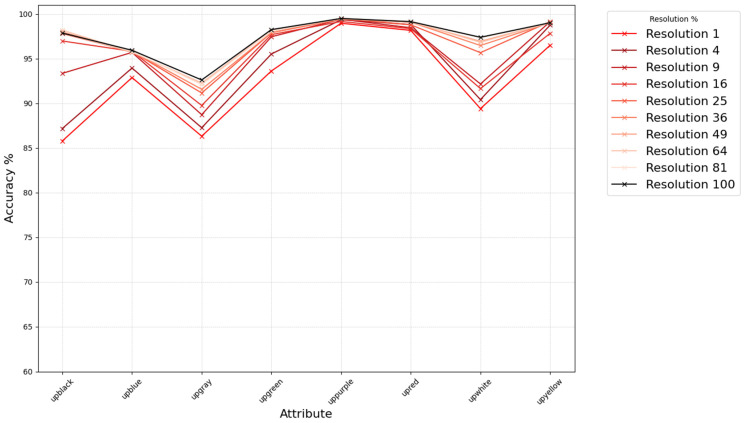
Accuracy of attribute color recognition for upper-body at the original and lower resolutions.

**Figure 7:**
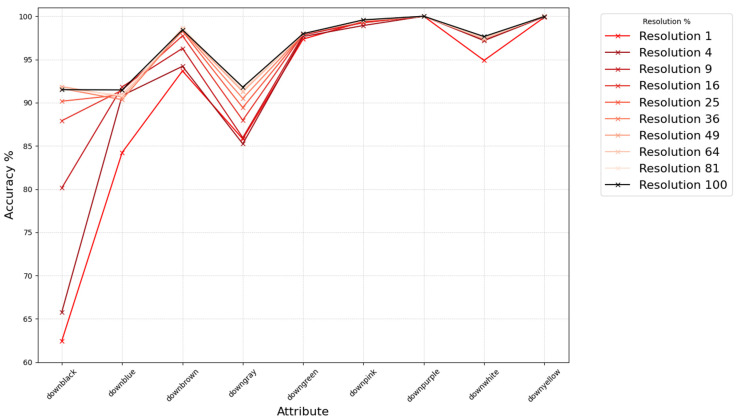
Accuracy of color attribute recognition for lower-body at the original and lower resolutions.

**Figure 8:**
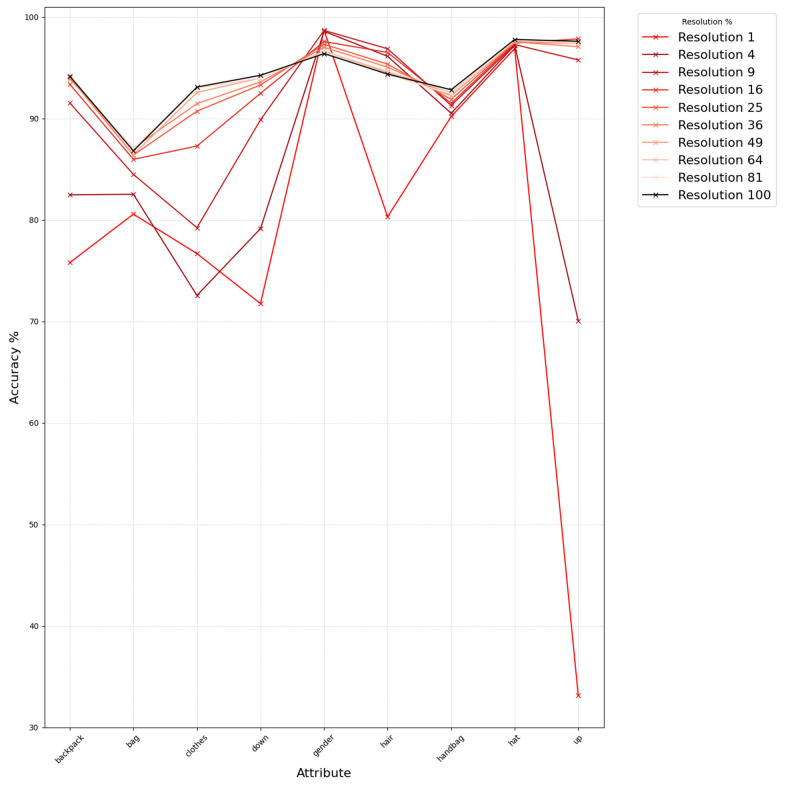
Accuracy of other attribute recognition for original and lower resolutions.

We verified whether similar trends hold for a real-world dataset based on the above findings. Using Market1501 [2,14], annotated with person attributes, we measured changes in attribute recognition under varying resolutions, unlike the previous experiment where we used rank-based analysis we directly classify each attribute (feature output from AMD [15]) based on a threshold of 0.5. We categorized attributes into the following three groups: upper-body color, lower-body color, and “other”, and observed how recognition accuracy changes as we progressively downscale the query images to different resolution levels (from the original size down to 1%), as shown in Figure 6, Figure 7 and Figure 8. From these figures, we see that the attribute recognition accuracy for each group is generally robust across resolutions. Upper-body color is particularly robust, while lower-body color is mostly robust except for certain attributes like “downblack” and “downblue.” The “other” group has a notably larger drop in accuracy as resolution decreases; interestingly, the “gender” attribute tends to become more accurate at lower resolutions, although overall it is less robust compared to the color-based groups.

To quantify these variations, we calculate the standard deviation of the accuracy in each group:

Upper-body color group—0.035568

Lower-body color group—0.065702

Other group—0.090174

The Other group (Figure 8) shows the highest variability, indicating that its accuracy declines more significantly under resolution degradation. Consequently, for our filtering approach, we select attributes from the upper- and lower-body color groups as our primary criteria.

### 3.4. Re-Identification Based on Attribute Information Using Filtering

When performing ReID on a low-resolution query image (captured by a drone or CCTV), we can leverage the attributes (such as the color of the upper and lower body) output by the explainable ReID model’s interpreter [15] to pre-filter the gallery images. By discarding irrelevant candidates upfront, we aim to boost both final ReID accuracy and inference speed. Algorithm 1 shows pseudocode for the proposed algorithm. Here, the “ReID Model” extracts deep-learning-based ReID embeddings, and the “Interpreter Model” infers human attributes (e.g., upper- or lower-body color). The method proceeds in four main steps:
**Algorithm 1.** Attribute based re-identification filtering pseudo codeInput: **Q** (query image), **G** (gallery images), ReID Model, Interpreter Model Output: Final ReID ranking list 1. // 1) Query embedding and attribute extraction 2. **F_Q** ← ReID_Model (**Q**)    // Query embedding (feature vector) 3. **A_Q** ← Interpreter (**Q**)     // Query attribute (e.g., upper/lower color) 4. // 2) Filter gallery by attribute 5. Filtered_G ← ∅ 6. for each image g in G do: 7.   **A_g** ← Interpreter (**g**)     // Extract attribute for g 8.   if Matches(**A_Q**, **A_g**) then  // e.g., same upper/lower color 9.     Filtered_G.add (**g**) 10. end for 11. // 3) Compute distances on filtered gallery 12. DistList ← [] 13. for each g in Filtered_G do: 14.   **F_g** ← ReID_Model (**g**)      // Gallery embedding 15.   **dist** ← || **F_Q**—**F_g** ||_2  // Euclidean distance 16.   DistList.add ((**g**, **dist**)) 17. end for 18. // 4) Sort and return top results 19. SortedList ← sort (DistList by dist ascending) 20. return SortedList       // used for Rank-1, Rank-5, Rank-10, etc.

The above pseudocode outlines the attribute-based ReID filtering procedure.

(1) Query embedding and attribute extraction: In lines 2–3, the algorithm extracts the query embedding **F**_**Q** from the query image **Q** (ReID_Model (**Q**)) and obtains the corresponding query attribute **A_Q** (Interpreter (**Q**)).
(2) Filter gallery by attribute: Lines 5–10 then interpret each gallery image **g** to obtain its attribute **A**_**g** (Interpreter (**g**)) and filter out images whose attributes do not match **A**_**Q**.
(3) Compute distances on filtered gallery: After the filtering step, lines 12–17 compute the Euclidean distance **dist** between the query embedding **F**_**Q** and each filtered gallery embedding **F**_**g**.
(4) Sort and return top results: Finally, lines 19–20 sort the resulting list of (**g**, **dist**) pairs by ascending distance and return the SortedList, which can be used to evaluate ReID performance (for example, Rank-1, Rank-5, or Rank-10).

**Figure 9:**
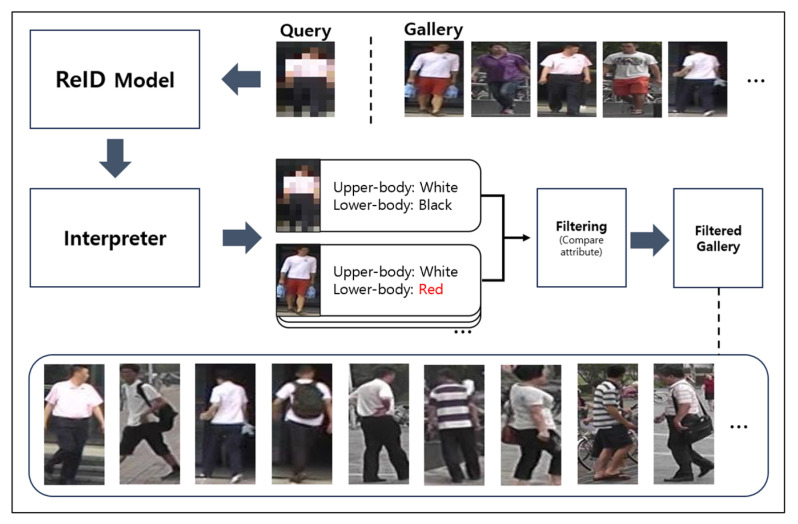
Attribute-based re-identification methods using filtering.

Figure 9 provides a schematic of the filtering for a query individual with a white upper-body color and black lower-body color. After filtering, the gallery retains only images of identities that share both those color attributes. By explicitly conducting attribute-based pre-filtering, we can swiftly remove gallery images that do not match the query’s upper- and lower-body colors even in low-resolution or noisy conditions and compare embeddings only among the remaining candidates, thereby improving both efficiency and accuracy.

## 4. Experiment on Enhancing Re-Identification Performance Through Filtering

**Figure 10:**
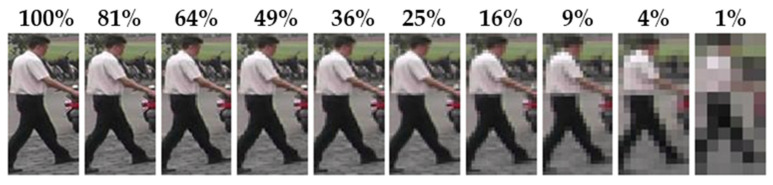
Example query images by resolution.

In this section, we validate whether the proposed method can improve performance when applied to low-resolution person images and describe the dataset configuration, experimental procedure, and evaluation. For the filtering experiments and performance assessment based on robust attribute information, we used the Market1501 official test protocol gallery (excluding the junk data that cannot be identified by ID) and the original query data. In addition, to examine ReID results under varying levels of resolution, we progressively scaled down the query images in both width and height by 10% increments, down to 1% of the original resolution. An example of these downscaled images is shown in Figure 10.

### 4.1. Experimental Methodology

We assess the extent to which the proposed filtering method can improve ReID accuracy across different resolutions by utilizing rank-based evaluation metrics, which are widely used to evaluate AI-based ReID models. In rank-based metrics, Rank-1 indicates how often the correct identity appears as the top-ranked result, whereas Rank-5 indicates how often the correct identity appears within the top 5 results, and so on.

**Figure 11:**
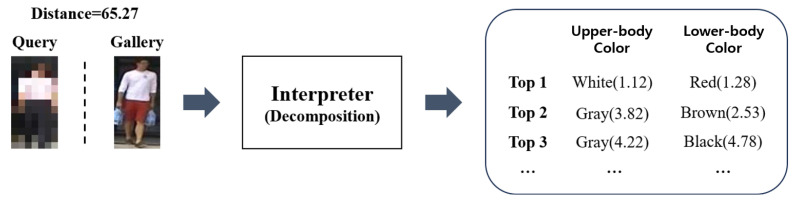
The process of inferring the color of clothing through the interpreter.

For the filtering criteria, we rely on the top-3 attribute values (determined by our interpreter model) for both the upper-body and lower-body colors, attributes that have been shown to be robust at low resolution. Figure 11 provides an example. During the explainable ReID process, the system decomposes the global scalar distance between query and gallery images according to individual attributes. Among these attributes, we select the strongest signals in upper- and lower-body colors as the filtering criteria, ranked in descending order.

To measure the performance of the proposed filtering method, we use the confusion matrix (TP, FP, TN, FN), commonly employed in classification tasks. When filtering based on low-resolution-robust attributes (upper-body and lower-body colors), these metrics are defined as follows:

TP: Cases that should be filtered out and are indeed filtered out.

FP: Cases that should be filtered out but are not actually filtered out.

TN: Cases that should not be filtered out and remain correctly retained.

FN: Cases that should not be filtered out but end up being filtered out.

Out of these, FNs are expected to have the most significant impact on ReID accuracy. A higher FN value implies that correct gallery images matching a given query have been mistakenly filtered out, lowering overall accuracy. Therefore, our experiments seek to identify the filtering criterion that minimizes FNs while confirming whether such a criterion leads to improved ReID accuracy.

### 4.2. Experiment and Results

Table 3, Table 4, Table 5 and Table 6 present the ReID performance and filtering algorithm metrics for different resolution levels and different filtering criteria. Each table shows the average ReID results obtained after applying filtering to the entire gallery for a specific resolution. The notation Top (n, m) denotes a criterion with the following values:

n is the number of top-ranked upper-body color attributes used.

m is the number of top-ranked lower-body color attributes used.

For example, Top (1, 1) means that the model uses only the single highest-scoring upper-body color and the single highest-scoring lower-body color to filter the gallery. Top (2, 3) means using the top 2 upper-body colors and the top-3 lower-body colors. If the gallery image is inferred to match the query’s clothing color among those top attributes, it remains in the gallery list; otherwise, it is discarded.

Images indicates how many gallery images remain (out of the original 15,913, excluding junk data) after applying the filtering. IDs is the number of unique identities (out of the original 750, excluding junk data) that remain in the filtered gallery.

Results at 1% resolution (Table 3): Under the Top (1, 1) criterion, filtering achieves the highest accuracy and even surpasses the baseline ReID without any filtering. While Top (3, 3) yields the lowest FN value, a low FN value does not necessarily translate into higher accuracy at this extreme resolution level.

Results at 4% resolution (Table 4): Again, Top (1, 1) yields the highest accuracy, although only Rank-10’s accuracy is notably higher than that of the baseline without filtering. While Top (3, 3) has the lowest FN value, as seen in Table 5, this does not automatically lead to improved accuracy.

Results at 9% resolution (Table 5): Under Top (3, 3), we see both the lowest FN value and the highest accuracy. Compared to pre-filtering, Rank-1 and Rank-5 accuracies both improve. Notably, Rank-1 accuracy increases from 69.33% to 74.88%, marking an increase of approximately 5.5 percentage points.

Results at 16% resolution (Table 6): Similarly to the 9% resolution, Top (3, 3) yields the lowest FN value and the highest accuracy. Rank-1, Rank-5, and Rank-10 accuracies all improve compared to no filtering. In particular, Rank-1 accuracy increases by 6.59 points, from 88.72% to 95.31%.

Considering all these results, for resolutions of 1% and 4% (Table 3 and Table 4), a low FN value did not necessarily lead to performance gains. On the other hand, Top (1, 1), despite having a high FN value, displayed slight improvements, though not substantial. These resolutions (1% and 4%) are extremely low (only 1% or 4% of the original image area), which likely explains the limited gains. In contrast, for 9% and 16% resolutions (Table 5 and Table 6), the criterion with the lowest FN value, namely Top (3, 3), consistently resulted in meaningful accuracy improvements over the unfiltered baseline.

Table 7 presents a comparative analysis of re-identification outcomes utilizing the AMD [15] model alongside the proposed methodology (ours) for query data categorized by resolution. The dimensional parameters of width and height were diminished to 10% and 20%, respectively, demonstrating the highest re-identification accuracy among the remaining resolutions when the filtering criteria were set to Top (3, 3), with the exception of the re-identification results for query data characterized by extremely low resolution, where the overall pixel reference resolution was reduced to 1% and 4%, correspondingly. Notably, it is evident that these outcomes indicate a significant enhancement in performance relative to pre-filtering conditions, particularly within the Rank 1 evaluation metric. It is observable that the application of the proposed filtering technique facilitates a substantial improvement in re-identification accuracy across diverse resolutions.

### 4.3. Analysis of Experimental Results

**Figure 12:**
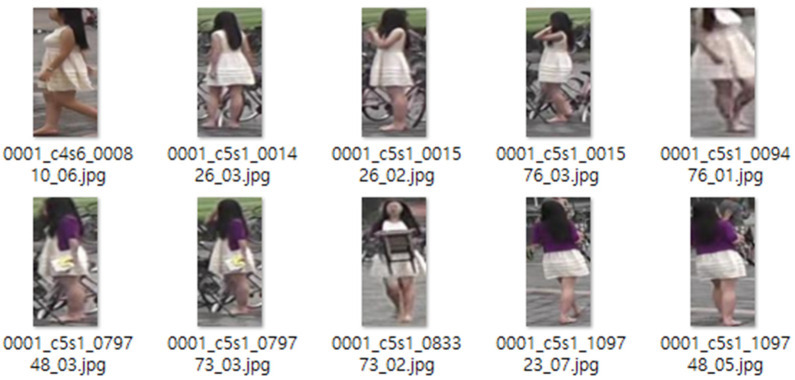
Gallery image of identity 0001 from the Market1501 dataset.

The empirical findings elucidated that the methodology posited in this manuscript enhances re-identification efficacy; however, the inferential outcomes for the predominant and subordinate colors yielded suboptimal results, wherein the value for the FN index did not attain 0 even with the Top (3, 3) filtering criteria, which minimizes FNs. To ascertain the underlying cause, the values of the primary and secondary color attributes of study [14], which conducted meticulous attribute labeling on the Market1501 dataset, were scrutinized. Consequently, it is primarily inferred that the rationale for the FN value not equating to zero is as follows:

The initial rationale is that individuals occasionally don garments of diverse hues, based on imagery from various galleries that exist under a singular identity. An illustration of this phenomenon can be seen in the gallery images for identity 0001, and the subsequent Figure 12 presents some of the gallery images for identity 0001 in the Market1501 dataset. The designation for identity 0001 is marked in white on both the upper and lower garments. Nevertheless, upon examining the actual clothing hues of the pertinent identity gallery images, depictions of individuals adorned in white upper- and lower-body clothing are observed, alongside images of them wearing purple cardigans. This discrepancy arises because the top and bottom colors of the query are designated as white, and in instances where a gallery is deduced in a color distinct from the query, filtration is enacted, resulting in scenarios where filtration occurs even for the same identity, which consequently leads to an escalation in the FN indicator value, indicative of a negative error. To address this quandary, when diverse colors are present for the same identity, it is feasible to implement a remedy by conducting multiple labeling for the color attribute or by performing labeling for each gallery image that exists for a singular identity.

**Figure 13:**
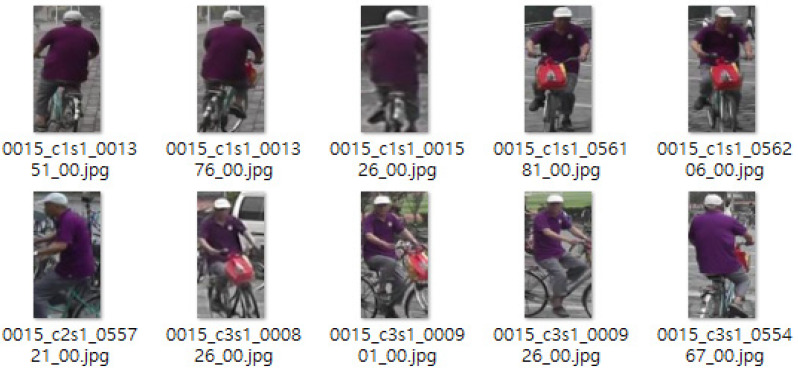
Gallery image of identity 0015 from the Market1501 dataset.

The second rationale is that the chromatic designation of the garment was merely executed inaccurately. Gallery depictions for identity 0015 serve as instances of this, and the subsequent Figure 13 presents some of the gallery depictions for identity 0015 within the Market1501 dataset. The predominant chromatic label for this identity is crimson, yet it can, in fact, be perceived as violet by the unaided eye. Consequently, when attribute designation of a specific identity is applied erroneously, the value for an error will escalate if filtration is conducted in comparison to the inference outcome.

**Figure 14:**
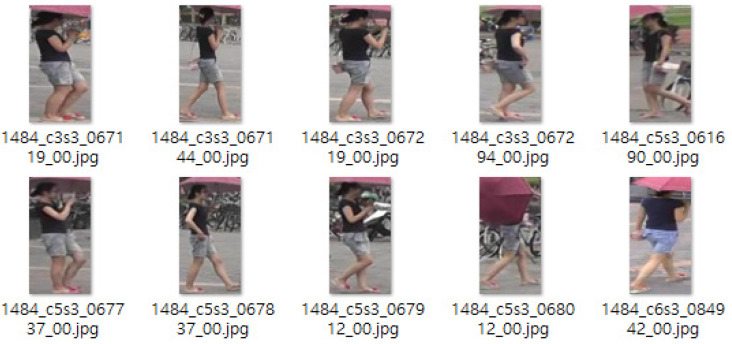
Gallery image of identity 1484 from the Market1501 dataset.

The tertiary cause pertains to situations in which the labeling corresponding to the hues of the upper and lower sections is accurately represented under normal circumstances; however, additional components within the visual representation may obstruct the accurate identification of the upper and lower sections. Illustrative examples can be found in the gallery images associated with identity 1484, and the subsequent Figure 14 showcases a selection of gallery images pertinent to identity 0015 within the Market1501 dataset. The figure under consideration is designated as black for the upper section and blue for the lower section; nevertheless, the complication arises from the presence of an image depicting a red umbrella within the region typically occupied by the upper section. In this instance, a scenario may arise in which the interpreter identifies the image as red, potentially resulting in an escalation of false error rates.

### 4.4. Performance Improvement in Re-Identification Computation Time

The filtering methodology is anticipated to enhance performance with respect to temporal efficiency. This enhancement arises from the diminution of time necessary for ReID, which is achieved by eliminating superfluous comparison subjects between queries and galleries. The temporal requirement during the re-identification phase, contingent upon the application of filtering, can be articulated through the subsequent equation.

Traditional Re-Identification Method: *T*^1^ = *f*(*N*, *K*) (1)

Re-Identification Method with Filtering: *T*^2^ = *f*(*N-F*, *K*) + *α* (2)

**Figure 15:**
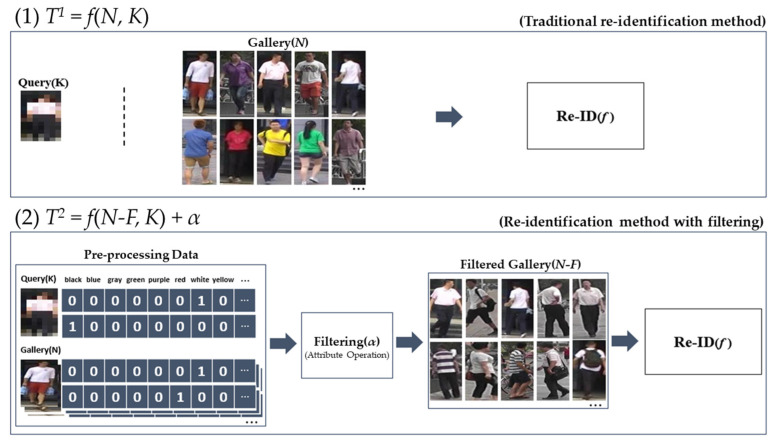
Computation time by re-Identification method.

In the aforementioned equation, *T* denotes the duration of computation pertinent to re-identification. *N* represents the quantity of gallery images, *K* signifies the total number of queried images, *F* indicates the count of filtered gallery images, and *α* refers to the computational time associated with the filtering process. Figure 15 serves as a visual representation elucidating the temporal requirements for the ReID methodology contingent upon the implementation of person attribute labeling and filtering processes.

Equation (1) delineates a conventional re-identification paradigm alongside the corresponding temporal requirements. *T*^1^, denoting the duration necessary for the ReID process, exhibits an increase that is directly proportional to both the quantity of gallery images (*N*) and the volume of query images (*K*). In instances where ReID is conducted repeatedly for the identical target, the requisite time escalates to *T*^1^ × *R*, wherein R is defined as the total number of iterations.

Equation (2) delineates the methodology and temporal requirements associated with the filtration of a gallery derived from pre-labeled data through a preprocessing phase, followed by the execution of ReID utilizing the filtered gallery (*N-F*). Given that α, representing the duration necessary for filtering, can be processed at an elevated velocity at the level of rudimentary bit computation, *T*^2^, the time requisite for re-identification subsequent to filtering, can achieve a speed enhancement of 1/((*N*-*F*)/*N*) times, in contrast to *T*^1^, relative to re-identification conducted prior to filtering. In instances where re-identification is performed iteratively for the identical target, denoting R as the iteration count, the duration extends to *T*^2^ × *R*, yielding a speed enhancement of 1/((*N*-*F*)/*N*)) × *R* when juxtaposed with the period prior to filtering. This indicates that a proportional enhancement in velocity can be realized in relation to the pre-filtering phase, contingent upon the frequency of re-identification iterations.

### 4.5. Discussion

In this discussion, we compare our filtering technique, which leverages attributes robust to low resolution in drone scenarios, with recent research on low-resolution ReID. We also address the potential problem of performance degradation in real-world surveillance scenarios, given that certain personal attributes are not permanent.

#### 4.5.1. Comparison: Super-Resolution (SR) Approach

Han et al. [8] and Chen et al. [18] attempted to improve ReID accuracy by restoring low-resolution images using SR. While SR can recover more high-resolution features at the pixel level, the additional network structures (e.g., Generators, Discriminators) impose heavy computation burdens in both training and inference. Although [8] adopts an adaptive SR to accommodate varying resolution conditions and [18] uses a multi-domain SR GAN for different environments, both remain expensive for real-time drone scenarios.

Our study shows that one can achieve up to a 6.59 percentage point improvement in Rank-1 accuracy at 16% resolution without any SR-based restoration. By filtering solely based on attribute information, we both reduce computational overhead and improve accuracy. Considering that real-world drones have constrained battery and network resources and that altitude and distance change frequently, omitting the high-resource demands of SR training and inference makes our method more practical for large-scale deployment.

#### 4.5.2. Comparison: Distillation·Cross-Resolution Approach

Munir et al. [19] proposed a method that uses images of different scales and feature distillation to reduce the feature vector gap across multiple resolutions. Similarly, cross-resolution ReID study [20] employ contrastive learning to correct low-resolution object recognition. Such distillation-based approaches often improve model accuracy but require more complex training procedures (multi-scale feature extraction, relational contrastive learning, etc.) and potentially additional data.

In contrast, our paper integrates a pre-trained ReID model with an explainable XAI interpreter to directly use person attributes (upper/lower color) for filtering, obviating the need for a separate distillation process or additional training. Consequently, in practical deployments, there is no need to re-train or adapt the model for multiple resolution scales; simple attribute-based filtering can alleviate the extremely low-resolution problem in aerial (drone) viewpoints. This is particularly advantageous for Fog/Edge Computing environments, where lightweight preprocessing at the node can reduce the number of gallery candidates and overall network traffic, facilitating real-time performance and system scalability.

#### 4.5.3. Potential Problem: Personal Attributes Are Not Permanent

In applying attribute-based filtering, we focus on upper- and lower-body colors, which tend to be relatively permanent or at least not subject to rapid change compared to accessories (e.g., bags, hats, umbrellas). Items like backpacks or umbrellas are easily removed or changed, which can cause inconsistencies and lower reliability for ReID in low-resolution settings. Indeed, if a subject briefly puts down a bag or takes off a hat, these transient attributes can disrupt ReID outcomes.

In contrast, the color of a person’s clothing (top/bottom), which we primarily rely on, remains consistent for a relatively long period and is still distinguishable even in extremely low-resolution drone images. As a result, we achieved a meaningful improvement of 6.59 percentage points in Rank-1 accuracy at 16% resolution with color attributes alone.

Nevertheless, in long-term tracking scenarios, an individual may change clothes or add new accessories, limiting the effectiveness of our current method. Potential solutions involve adding more fundamental and stable biometric or behavioral features, such as body shape or gait, or using an online update mechanism for attributes that dynamically adapt to changes in clothing. We leave these expansions for future work, noting that our study demonstrates the practical potential of attribute-based filtering to mitigate frequent low-resolution challenges in aerial surveillance.

## 5. Integration with Fog/Edge Computing Infrastructure

**Figure 16:**
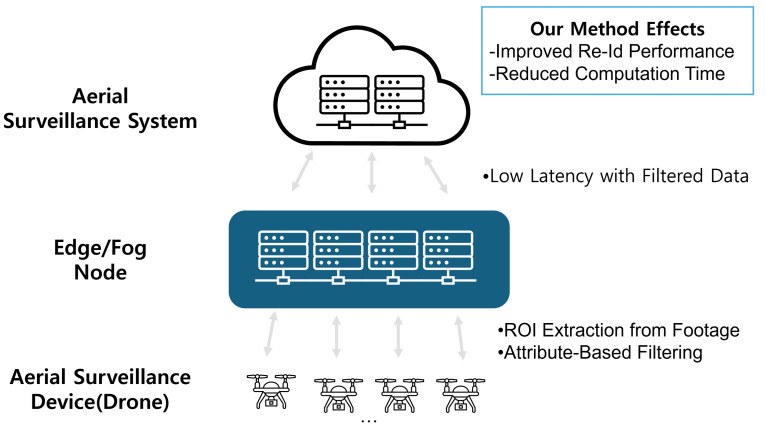
Diagram of strategies for utilizing Fog and Edge Computing.

Drone-based aerial surveillance systems often face constraints such as limited battery life and the computing power of the drones themselves, as well as physical separation from the control server, resulting in network latency. As a solution, recent research has drawn attention to Fog/Edge Computing, which performs certain portions of data preprocessing and attribute filtering (e.g., ROI extraction, DRL-based offloading) at the network Edge or Fog Nodes instead of the central server [5,9,10]. This approach alleviates communication delays and computational overhead by only sending essential information to the cloud. Figure 16 provides an overview of how the proposed person ReID framework can be integrated into a Fog/Edge Computing environment for a drone-based aerial surveillance system.

### 5.1. Strategies for Integration Fog and Edge Computing

Because drones have restricted battery and computational resources, and because of their mobility and the resulting latency in data transmission [9], an effective approach is to distribute lightweight preprocessing and attribute filtering to Edge/Fog Nodes, transmitting only essential data (or partial results) to the cloud. Recently, DRL-based offloading techniques have also been proposed to dynamically minimize computational delay in a wireless power environment [5]. This form of distributed processing plus intelligent offloading can mitigate the constraints posed by low-resolution drone imagery and limited bandwidth, enhancing real-time capabilities.

#### 5.1.1. Aerial Surveillance Device (Drone)

(1) Video Capture: The drone acquires live video frames using an onboard camera sensor. Given the significant limitations in battery and hardware resources, it performs only minimal preprocessing (e.g., ROI detection) to reduce power consumption.

(2) Data Transmission: The drone transmits only the ROI (or metadata from selected frames) to the Edge/Fog Node, thus conserving both network bandwidth and the drone’s battery. By avoiding the transmission of the entire original video, unnecessary communication overhead is substantially reduced [9].

#### 5.1.2. Edge/Fog Node

(3) Attribute-Based Filtering: Upon receiving the drone video or ROI data, the Edge/Fog Node applies a lightweight object detection model to further refine the region of interest related to humans. It then performs robust attribute filtering (e.g., upper/lower-body color) and partial ReID inferencing to reduce the set of candidate images.

(4) ReID Model Inference: By leveraging the stable attributes robust to low resolution, the Edge/Fog Node narrows down gallery images and applies the ReID model to infer person ID and attributes.

(5) Result Transmission: Only essential results (e.g., identity, key attribute information) and selected features are then transferred to the cloud, minimizing the volume of data sent over the network.

#### 5.1.3. Aerial Surveillance System (Cloud)

(6) Aggregated Analysis and Visualization: The cloud server integrates and analyzes the results collected from multiple Edge/Fog Nodes, offering a unified view for aerial surveillance, monitoring, or control.

(7) Data Storage and Integration: To facilitate long-term storage, the cloud preserves both the processed video data and inference results, making them available for subsequent model retraining or advanced analytics.

(8) Model Retraining and Management: By leveraging large-scale GPU/TPU clusters, the cloud can regularly retrain the model, tune hyperparameters, and update the attribute information, before redeploying the improved model to Edge/Fog Nodes to sustain or enhance ReID performance.

### 5.2. Potential Benefits from Fog/Edge Integration

A distributed architecture that spans the drone, Edge/Fog Nodes, and the cloud offers the following potential advantages:

Improved ReID Accuracy in Low-Resolution Settings: Even when the drone video is of low resolution, leveraging robust attributes (e.g., top/bottom color) to filter out unrelated candidates can enhance ReID accuracy. By performing attribute filtering and preliminary screening at the edge, only critical frames or metadata need be transmitted to the cloud, thereby reducing false positives and missed detections.

Reduced Computation and Faster Response: Since the gallery size is significantly reduced prior to the main ReID process, the computational overhead at each step also diminishes. With less data to analyze and less network traffic, the system can achieve near real-time or real-time performance.

Mitigation of Network Traffic and Latency: Instead of transmitting full-resolution videos to the cloud, the Edge/Fog Node only sends the essential data (attribute information, IDs). This relieves network congestion, especially in large-scale deployments with multiple drones operating simultaneously. Fog-level distributed processing helps prevent communication bottlenecks.

Scalability and Operational Efficiency: The system can be flexibly scaled out by adding more Edge/Fog Nodes as needed. When the model is updated or retrained in the cloud, the new version can be swiftly deployed across Edge/Fog Nodes, keeping the ReID performance up-to-date without shutting down the entire system.

### 5.3. Challenges in Integrating the Proposed Method with Fog/Edge Computing

While integrating the proposed attribute-based ReID filtering method into a Fog/Edge environment can yield advantages in real-time performance and network efficiency, several practical issues must be taken into account:

#### 5.3.1. Implementation Approach: Monolithic vs. Microservices

Monolithic Architecture: Deploying all functionalities (ROI extraction, attribute filtering, ReID model inference, etc.) on a single edge server may minimize initial implementation and maintenance costs. However, should that edge server fail, the entire system is disrupted. Moreover, as the number of drones grows or the surveillance area expands, the scalability of a single monolithic server can become a major limitation.

Microservices Architecture: Separating functionalities such as attribute filtering, ReID inference, and XAI visualization into distinct services and distributing them across multiple Edge/Fog Nodes increases scalability (scale-out) and fault tolerance. At the same time, it adds complexity in terms of service-to-service communication (REST, gRPC) and orchestration platforms (Kubernetes, Docker Swarm).

Deciding on the appropriate architecture depends on available development and operational resources, maintenance capacity, and overall system goals. Although microservices generally provide better scalability and fault tolerance, they demand container orchestration or a DevOps/MLOps framework, leading to higher initial deployment and maintenance costs. Consequently, a phased approach can be practical: begin with a monolithic architecture at the proof-of-concept stage for quick validation, then transition to a microservices architecture once the scale of operation increases.

#### 5.3.2. Where the Proposed Methodology Could Be Provisioned?

Resource and Network Limitations at the Edge/Fog Node: If the Edge environment has limited CPU/GPU capacity for ReID model inferencing, or if the wireless connection (5G, Wi-Fi, etc.) is unstable, one may need to employ model compression (e.g., pruning, quantization) or only perform partial filtering. In cases where power or cooling resources are severely restricted, additional functionality might need to be offloaded to nodes with stable energy supplies or to a nearby data center.

Latency vs. Bandwidth Trade-off: Placing the ReID functionality in an Edge Node located physically close to the drone can reduce communication latency. On the other hand, if high-resolution footage must be sent to that node, bandwidth congestion could occur. An effective balance between these factors is essential.

Traffic Surge Scenarios: Traffic volumes may spike suddenly along drone flight paths or during events such as large gatherings or sporting occasions. In such cases, a DRL-based offloading policy can be used to efficiently allocate resources. If resources become too constrained or the network connection becomes unstable, the system may prioritize only essential attributes (e.g., upper- or lower-body color) under low-resolution conditions, while temporarily suspending advanced analyses (XAI, detailed attributes).

By comprehensively accounting for these integration strategies and challenges, one can build a surveillance system capable of effectively processing and analyzing low-resolution drone footage in real or near-real time.

## 6. Future Work

Moving forward, there are several avenues we intend to explore in order to further enhance the robustness and applicability of our approach. While this paper primarily addresses upper-body and lower-body colors, real-world surveillance settings often involve sudden changes in attire or the use of accessories, such as bags, umbrellas, and hats. We therefore plan to expand beyond color-based attributes by integrating more robust features like body shape and gait patterns and by adopting an online mechanism to dynamically update attribute labels when a person’s clothing changes.

We also seek to mitigate the domain gap that arises when transitioning from synthetic to real-world drone footage. Although synthetic data provides precise control over factors such as distance, altitude, and camera angle, inevitable differences in texture, lighting, and sensor noise can limit the direct applicability of trained models to real-world conditions. To address this, we will investigate advanced post-processing and domain-adaptation strategies, ensuring that our models can effectively generalize to diverse drone-captured images.

Another focal area is extending ReID to extreme low-resolution scenarios. In highly elevated or wide-area surveillance contexts, individuals may occupy only a handful of pixels, posing significant challenges to feature extraction. We intend to examine cross-resolution frameworks and knowledge-distillation techniques that can further improve the performance of our attribute-filtering approach in these demanding environments.

Lastly, we aim to refine Fog/Edge deployment strategies to better balance on-board (edge) versus cloud-level processing. By incorporating additional modules such as DRL-based offloading or federated learning we hope to design more adaptive systems that autonomously adjust to fluctuating network bandwidth, computational resources, and the evolving demands of aerial surveillance. Our ultimate goal is to build a truly flexible and efficient ReID framework capable of robust, real-time performance across a range of operational conditions.

## 7. Conclusions

In this paper, we addressed the challenge of significant performance degradation in person re-identification (ReID) systems when dealing with low-resolution drone imagery. By systematically generating synthetic datasets using Unreal Engine 5 and AirSim, we established a controlled environment to investigate how drone altitude and distance affect visual resolution and, consequently, ReID accuracy. Through an XAI framework, we identified specific human attributes—particularly upper- and lower-body colors that remain robust even with severe downsampling. Leveraging these stable attributes, we proposed a lightweight filtering method that discards irrelevant gallery candidates and thus enhances both ReID accuracy and computational efficiency.

Experimental results on the Market1501 dataset demonstrated that our attribute-based filtering approach can boost Rank-1 accuracy by up to 6.59 percentage points at a 16% resolution scale without resorting to computationally expensive Super-Resolution techniques. Moreover, we discussed how our method can be seamlessly integrated into Fog/Edge Computing infrastructures, enabling real-time processing of large-scale drone imagery with reduced latency and network overhead. Overall, our findings highlight the potential of attribute-guided filtering to mitigate resolution-related challenges, paving the way for more efficient and accurate drone-based surveillance systems.

## Figures and Tables

**Table 1:** Personal attribute details for explainable re-identification model.

Attribute	Representation in File	Label
gender	gender	male (1), female (2)
hair length	hair	long sleeve (1), short sleeve (2)
length of lower-body clothing	down	long lower body clothing (1), short (2)
type of lower-body clothing	clothes	dress (1), pants (2)
wearing hat	hat	no (1), yes (2)
carrying backpack	backpack	no (1), yes (2)
carrying bag	bag	no (1), yes (2)
carrying handbag	handbag	no (1), yes (2)
8 colors of upper-body clothing	upblack, upwhite, upred, uppurple, upyellow, upgray, upblue, upgreen	no (1), yes (2)
9 colors of lower-body clothing	downblack, downwhite, downpink, downpurple, downyellow, downgray, downblue, downgreen, downbrown	no (1), yes (2)

**Table 2:** Summary of synthetic datasets used in experiments.

Attribute	Value	Count
Human (including 26 attributes)	10 assets	10
Horizontal angle	0~330° (step = 30)	12
Distance	6 m~36 m(step = 1)	31
Total image	3720	

**Table 3:** Re-identification results after filtering at 1% resolution.

Filtering Criterion	Images	IDs	TP	FP	TN	FN	Rank1	Rank5	Rank10
Top (1, 1)	859	104	14,878	508	350	174	0.65%	2.55%	5.67%
Top (1, 2)	1680	188	14,112	1275	405	119	0.33%	1.07%	2.2%
Top (1, 3)	2263	219	13,547	1839	423	102	0.21%	0.71%	1.28%
Top (2, 1)	1834	199	13,936	1451	383	142	0.3%	1.57%	2.97%
Top (2, 2)	3586	352	12,244	3143	443	82	0.12%	0.56%	1.48%
Top (2, 3)	4807	407	11,042	4344	463	62	0.06%	0.33%	0.89%
Top (3, 1)	2337	236	13,442	1945	392	132	0.24%	0.86%	1.81%
Top (3, 2)	4564	413	11,277	4110	454	71	0.03%	0.36%	0.71%
Top (3, 3)	6116	474	9746	5640	475	50	0%	0.15%	0.42%

**Table 4:** Re-identification results after filtering at 4% resolution.

Filtering Criterion	Images	IDs	TP	FP	TN	FN	Rank1	Rank5	Rank10
Top (1, 1)	1186	137	14,541	852	334	187	6.74%	14.25%	19.98%
Top (1, 2)	2311	224	13,473	1919	391	128	6.03%	12.11%	16.86%
Top (1, 3)	2910	257	12,894	2498	412	108	5.91%	11.37%	14.88%
Top (2, 1)	2505	256	13,254	2138	367	152	6.32%	12.62%	17.13%
Top (2, 2)	4867	404	10,958	4435	432	88	5.55%	11.01%	14.13%
Top (2, 3)	6098	460	9749	5643	455	65	5.58%	10.36%	13.42%
Top (3, 1)	3155	300	12,616	2776	378	141	6.03%	11.64%	15.91%
Top (3, 2)	6122	467	9715	5677	445	74	5.34%	10.33%	13.24%
Top (3, 3)	7051	511	8809	6625	426	52	1.69%	3.5%	4.66%

**Table 5:** Re-identification results after filtering at 9% resolution.

Filtering Criterion	Images	IDs	TP	FP	TN	FN	Rank1	Rank5	Rank10
Top (1, 1)	2966	268	12,748	2648	318	199	59.77%	69.03%	72.57%
Top (1, 2)	4625	349	11,166	4230	395	120	67.4%	76.66%	79.66%
Top (1, 3)	5305	384	10,509	4886	418	97	69.78%	78.56%	81.71%
Top (2, 1)	5670	519	10,084	5311	359	157	63.48%	73.22%	76.75%
Top (2, 2)	8800	526	7042	8354	446	70	72.18%	81.5%	84.68%
Top (2, 3)	10,119	576	5749	9646	472	43	74.08%	83.11%	86.37%
Top (3, 1)	6373	457	9391	6004	369	147	64.73%	74.14%	77.76%
Top (3, 2)	9914	573	5940	9455	459	57	73.13%	82.31%	85.39%
Top (3, 3)	11,447	628	4435	10,961	486	30	74.88%	83.7%	86.76%

**Table 6:** Re-identification results after filtering at 16% resolution.

Filtering Criterion	Images	IDs	TP	FP	TN	FN	Rank1	Rank5	Rank10
Top (1, 1)	4009	332	11,717	3682	327	187	75.62%	79.25%	80.29%
Top (1, 2)	5980	413	9822	5576	404	110	84.8%	87.56%	88.51%
Top (1, 3)	6273	447	9098	6300	423	90	87.08%	89.58%	90.47%
Top (2, 1)	6782	467	8985	6412	369	144	81.7%	85.51%	86.37%
Top (2, 2)	10,075	5779	5779	9619	457	58	91.69%	94.21%	94.8%
Top (2, 3)	11,382	612	4495	10,902	479	34	93.74%	95.81%	96.47%
Top (3, 1)	7444	503	8332	7065	378	135	83.73%	87.23%	88.15%
Top (3, 2)	11,074	611	4791	10,606	468	46	93.44%	95.75%	96.32%
Top (3, 3)	12,554	661	3336	12,061	492	23	95.31%	97.15%	97.65%

**Table 7:** Apply filtering by resolution and compare results.

Resolution	Rank 1			Rank 5		
	AMD	Ours	Compare (+/−)	AMD	Ours	Compare (+/−)
1%	0.06%	0.65%	+0.59%	0.24%	2.55%	+2.31%
4%	7.73%	6.74%	−0.99%	14.7%	14.25%	−0.45%
9%	69.33%	74.88%	+5.55%	82.81%	83.7%	+0.89%
16%	88.72%	95.31%	+6.59%	96.02%	97.15%	+1.13%
25%	93.35%	96.97%	+3.62%	97.68%	98.13%	+0.45%
36%	93.71%	97.48%	+3.77%	97.74%	98.19%	+0.45%
49%	94.57%	97.57%	+3.00%	97.92%	98.28%	+0.36%
64%	94.92%	97.80%	+2.88%	98.1%	98.43%	+0.33%
81%	94.86%	97.71%	+2.85%	98.04%	98.22%	+0.18%
100%	95.13%	97.65%	+2.52%	98.19%	98.28%	+0.09%

## Data Availability

The original contributions presented in this study are included in the article. Further inquiries can be directed to the corresponding author.

## References

[1] Government of Korea Installing and Operating CCTV in Public Institutions. https://www.index.go.kr/unity/potal/main/EachDtlPageDetail.do?idx_cd=2855.

[2] Zheng L., Shen L., Tian L., Wang S., Wang J., Tian Q. Scalable person re-identification: A benchmark. Proceedings of the IEEE International Conference on Computer Vision.

[3] Wei L., Zhang S., Gao W., Tian Q. Person transfer gan to bridge domain gap for person re-identification. Proceedings of the IEEE Conference on Computer Vision and Pattern Recognition.

[4] Cao Y., He Z., Wang L., Wang W., Yuan Y., Zhang D., Zhang J., Zhu P., Van Gool L., Han J. VisDrone-DET2021: The vision meets drone object detection challenge results. Proceedings of the IEEE/CVF International Conference on Computer Vision.

[5] Zheng K., Jiang G., Liu X., Chi K., Yao X., Liu J. (2023). DRL-based offloading for computation delay minimization in wireless-powered multi-access edge computing. IEEE Trans. Commun..

[6] Ye M., Shen J., Lin G., Xiang T., Shao L., Hoi S.C. (2021). Deep learning for person re-identification: A survey and outlook. IEEE Trans. Pattern Anal. Mach. Intell..

[7] Dilshad N., Hwang J., Song J., Sung N. Applications and challenges in video surveillance via drone: A brief survey. Proceedings of the 2020 International Conference on Information and Communication Technology Convergence (ICTC).

[8] Han K., Huang Y., Song C., Wang L., Tan T. (2021). Adaptive super-resolution for person re-identification with low-resolution images. Pattern Recognit..

[9] Bonomi F., Milito R., Zhu J., Addepalli S. Fog computing and its role in the internet of things. Proceedings of the First Edition of the MCC Workshop on Mobile Cloud Computing.

[10] Li T., Sahu A.K., Talwalkar A., Smith V. (2020). Federated learning: Challenges, methods, and future directions. IEEE Signal Process. Mag..

[11] Epic Games Unreal Engine 5. https://www.unrealengine.com.

[12] Shah S., Dey D., Lovett C., Kapoor A. Airsim: High-fidelity visual and physical simulation for autonomous vehicles. Proceedings of the Field and Service Robotics: Results of the 11th International Conference.

[13] Adadi A., Berrada M. (2018). Peeking inside the black-box: A survey on explainable artificial intelligence (XAI). IEEE Access.

[14] Lin Y., Zheng L., Zheng Z., Wu Y., Hu Z., Yan C., Yang Y. (2019). Improving person re-identification by attribute and identity learning. Pattern Recognit..

[15] Chen X., Liu X., Liu W., Zhang X.-P., Zhang Y., Mei T. Explainable person re-identification with attribute-guided metric distillation. Proceedings of the IEEE/CVF International Conference on Computer Vision.

[16] Zheng L., Yang Y., Hauptmann A.G. (2016). Person re-identification: Past, present and future. arXiv.

[17] Chen Y.-C., Zhu X., Zheng W.-S., Lai J.-H. (2017). Person re-identification by camera correlation aware feature augmentation. IEEE Trans. Pattern Anal. Mach. Intell..

[18] Chen S., Wu H., Chen Y. Multi-Domain Image Super-Resolution Generative Adversarial Network for Low-Resolution Person Re-Identification. Proceedings of the 2021 40th Chinese Control Conference (CCC).

[19] Munir A., Lyu C., Goossens B., Philips W., Micheloni C. Resolution based feature distillation for cross resolution person re-identification. Proceedings of the IEEE/CVF International Conference on Computer Vision Workshops (ICCVW).

[20] Zhang K., Ge S., Shi R., Zeng D. (2023). Low-Resolution Object Recognition with Cross-Resolution Relational Contrastive Distillation. IEEE Trans. Circuits Syst. Video Technol..

[21] Porrello A., Bergamini L., Calderara S. Robust re-identification by multiple views knowledge distillation. Proceedings of the Computer Vision–ECCV 2020: 16th European Conference.

[22] Yu X., Gong Y., Jiang N., Ye Q., Han Z. Scale match for tiny person detection. Proceedings of the IEEE/CVF Winter Conference on Applications of Computer Vision.

[23] Li H., Ye M., Du B. Weperson: Learning a generalized re-identification model from all-weather virtual data. Proceedings of the 29th ACM International Conference on Multimedia.

[24] Zhang T., Xie L., Wei L., Zhuang Z., Zhang Y., Li B., Tian Q. Unrealperson: An adaptive pipeline towards costless person re-identification. Proceedings of the IEEE/CVF Conference on Computer Vision and Pattern Recognition.

[25] Ma S., Yuan Z., Wu Q., Huang Y., Hu X., Leung C.H., Wang D., Huang Z. (2023). Deep into The Domain Shift: Transfer Learning through Dependence Regularization. IEEE Trans. Neural Netw. Learn. Syst..

[26] Delussu R., Putzu L., Fumera G. On the effectiveness of synthetic data sets for training person re-identification models. Proceedings of the 2022 26th International Conference on Pattern Recognition (ICPR).

[27] Wang J., Feng Z., Chen Z., George S., Bala M., Pillai P., Yang S.-W., Satyanarayanan M. Bandwidth-efficient live video analytics for drones via edge computing. Proceedings of the 2018 IEEE/ACM Symposium on Edge Computing (SEC).

[28] Khaldi K., Nguyen V.D., Mantini P., Shah S. Unsupervised person re-identification in aerial imagery. Proceedings of the IEEE/CVF Winter Conference on Applications of Computer Vision.

[29] Liu X., Chen A., Zheng K., Chi K., Yang B., Taleb T. (2024). Distributed Computation Offloading for Energy Provision Minimization in WP-MEC Networks with Multiple HAPs. IEEE Trans. Mob. Comput..

[30] Li H., Xiong K., Lu Y., Gao B., Fan P., Letaief K.B. (2021). Distributed design of wireless powered fog computing networks with binary computation offloading. IEEE Trans. Mob. Comput..

[31] He H., Zhou C., Huang F., Shen H., Li S. (2024). Energy-Efficient Task Offloading in Wireless-Powered MEC: A Dynamic and Cooperative Approach. Mathematics.

